# Vital Force

**Published:** 1878-08

**Authors:** A. R. Shank

**Affiliations:** President of the Cayuga County Medical Society


BUFFALO

### Medical and Surgical Journal.

VOL. XVIII. AUGUST, 1878. NO. 1.

### Original Communications.

—:o:—

*ART. I.—Vital Force.* An Address by Dr. A. R. SHANK, President of the Cayuga County Medical Society, given at the Annual Meeting of the Society, June 12th, 1878.

Ladies and Gentlemen of The Medical Society of the county of Cayuga: In concluding the performance of those duties which devolve upon your presiding officer, I am required by our by-laws to deliver before this Society an address upon some subject pertaining to either Medicine or Surgery.

I apprehend that whatever pertains to or holds a definite relation towards any physiological action, will be regarded by you as a proper subject for consideration on this occasion.

Some two years ago I had the honor of reading before the Auburn City Medical Society an Essay, the subject of which was The Correlation of Physical and Vital Forces. Owing to the fact that that essay was sadly incomplete in its conclusions, I then promised the members of that society, that at some future time I would pursue the subject a step further, in order to show that in the exercise of our mental, as in all other bodily functions, the same relations with the physical forces are found to exist. Unless, therefore, I was very unfortunate in adopting a theme for the

79282

paper just alluded to, in asking your attention at this time to a kindred subject, I shall not only comply with the requirements of our by-laws, but also fulfill my promise to the members of the City Medical Society.

Granting that the correlation of those forces which are by all regarded as purely physical, is as firmly established as the law that governs the momentum and velocity of falling bodies,—a fact which none present will dispute,—we may immediately proceed to the discussion of the subject before us, namely, Vital Force.

If we desire to rear a sound fabric of biology, we must begin our study of life with the observation of its humblest and most universal manifestations and from thence conduct our investigations by strictly scientific methods. The most universal and fundamental characteristic of life is that mode of vital activity which manifests itself in the evolution of the germ into a complete organism, repeating the type of its parents, and the subsequent maintenance of that organism in its integrity at the expense of materials derived from external sources.

Let us then, in pursuance of our subject, begin with the consideration of the nature and source of the force or forces by which inorganic binary compounds are raised to the grade of complex proximate principles and so combined as to produce a duplicate of the parent organism. The prevailing opinion was, until lately, that this power was inherent in the germ; which was supposed to derive from its parents not only its material substance but also a germ-force, in the exercise of which it not only built itself up in the likeness of its parents and preserved the integrity of its complete fabric, but also imparted a fraction of that force to each of its progeny.

Thus all the organizing force required to build up the vast fabrics of the mighty forest giants of the Caliveras valley and all their succeeding progeny, must have been contained in a minute particle of matter, only discernable by means of the closest microscopic inspection, and weighing, perhaps, no more than the thousandth of a grain. So, too, by that hypothesis, the germ-force which has organized the bodies of the whole human race must have been contained in that of Adam, its common progenitor. A more complete

*reductio ad absurdum* can scarcely be brought against any hypothesis, and it is manifest that we must look to some source without the organism for that force which is constantly expended in the construction and maintenance of living fabrics.

However the germ may have come in possession of its endowment, its office appears to be that of a directing agent; its function resembling that of a master builder having charge of workmen who labor under his direction.

If we extensively survey the phenomena of life as exhibited in both the animal and vegetable kingdoms, and carefully observe those influences which invariably accelerate or retard (though never preventing) growth and development, we can not but conclude that heat—the influence of which upon both structure and function is so marked—is the actual constructive force which performs the work of organization of all animal and vegetable tissue.

I now desire to direct your attention to some of those phenomena which appear best to illustrate the mode in which heat acts upon the living organism, promoting its growth and development. Let us commence with a seed of the vegetable kingdom. It consists of an embryo which, owing to parental influence, is already advanced to a certain stage of development, and which, by the same agency, is supplied with a store of nutriment, laid up for use in its further evolution.

Now, under suitable conditions, this seed will remain for an indefinite period of time in a state of inactivity; but the moment it is exposed to air and moisture and subjected to a higher temperature, it either germinates or decays. Which of these two opposite changes shall take place under this increment of heat-force appears to depend upon the loss or retention by the embryo of its vital endowment. If that endowment has been retained, and the requisite supply of air, moisture and heat be furnished, certain chemical activities will immediately ensue. The insoluble starch which surrounds the embryo is, by the agency of a ferment called diastase, brought into a soluble form called dextrine, and then, if the conditions be continued, into sugar. The dextrine and sugar combining with the albuminous and oily compounds stored in the seed forms protoplasm, which is the material basis out of which all animal and vegetable tissues immediately arise.

But in the conversion of the insoluble starch into sugar and also, as is probable, by a further change of a portion of the sugar, a large quantity of carbon is eliminated by combining with oxygen of the air, which combination is necessarily attended with the disengagement of heat—as may be seen in the process of malting.

Now since it is invariably true that the rate of growth and development in the germinating embryo is (within certain limits) closely correspondent with and in a direct ratio to the quantity of heat supplied it must be regarded as highly probable that the heat disengaged during the process of germination, concurring with that derived from external sources, constitutes the force employed in the evolution of the germ.

Thus far the condition and behavior of the embryo animal resembles that of the vegetable. But when the plant has reached a more advanced stage of development it acquires the ability to form the materials necessary for the extension of its fabric, instead of receiving its supply, as at first, from some previous agency. The tissues of the colored surfaces of its leaves and stems, when acted on by light, have the power to generate, at the expense of carbonic acid water and ammonia, various organic compounds, such as chlorophile, starch and albumen, a portion of which is appropriated to the formation of new tissue, while another portion is stored away within the cavities of the tissue already formed, when it may serve for the nutrition of animals using them for food, while still another portion undergoes a retrograde metamorphosis, thereby liberating such a measure of heat, (or constructive force,) as may be necessary to maintain the specific temperature of the organism whenever subjected to a measure of external heat less than its own, and whereby, also, by a progressive metamorphosis, another portion is raised to the level of organized living tissue.

The influence of light upon vegetable organisms appears to be exerted in bringing about what may be considered a higher mode of chemical combination; but that the appropriation, by the more fully developed plant, of the material thus produced and its conversion into living organized tissue, is as dependent on heat as ar those earlier structural changes which occur during the period of germination, is obvious.

When instead of yielding any portion of its tissue for the nutrition of animals the entire fabric undergoes a retrograde metamorphosis, whether by slow decay or ordinary combustion, it gives back to the inorganic world, not only every particle of inorganic matter contained within its fabric, but also the exact equivalents of light and heat that were employed in elevating it to the level of organized living tissue. Hence we are justified in the conclusion, that the correlation between heat and the organizing force of plants is not less intimate than that existing between heat and motion or other forms of physical force.

In the ovum of the animal as in the seed of the plant, a supply of organic compounds is provided; and that supply is suitable in kind and sufficient in quantity for the development of the contained embryo until it reaches a stage of advancement when the young animal becomes able to ingest material for its subsistence. But that the animal ovum is as dependent upon heat as a constructive force in its development as is the germinating seed, is proved by the fact that the eggs of the orchard caterpillar may be made to produce a brood as readily in the winter as at that season of the year when, as is ordinarily the case, their emersion occurs at a time when the leaves suitable for their food put forth.

The stage of development at which animals of different tribes come from their ova are widely dissimilar. Some commence their independent existence at a stage of development which, as compared with the adult, is as if a human fetus should be thrown upon the world within a few weeks after conception. We see in the flesh-fly a typical example of this. When first entered upon its larva state it is an extremely simple and minute worm, nearly incapable of movement but possessed of extraordinary voracity. Owing to its favorable surroundings it is as well provided with alimentary organic compounds as if supplied with a supplimentary yolk. By availing itself of this alimentary provision it soon grows to many times its former size without, however, making any perceptible advance in development. But soon, having laid up within its tissues a store of additional material it passes into a state which, as far as outward appearances indicate, is one of torpor, but which is really one of great developmental activity. For it is during this pupa state that those parts are evolved which characterize the perfect insect. And since during this purely developmental stage of its existence, it rapidly and invariably loses a portion of its weight and also exhales carbonic acid and water in amount excessively disproportionate to the heat liberated from its body, we are left to no other conclusion than that there is a retrograde metamorphosis of a part of the store of materials laid up during the larva state, and that a portion of the force released during this retrogressive change is expressed in the form of heat, while, that remaining portion which does not make itself thus manifest is consumed in the process of progressive evolution.

There are insects whose lives are of but short duration, during which they take no food whatever. All their vital activities must therefore depend upon the store of material ingested during their larva state. The sole object of their existence appears to be the generative act, after performing which they almost immediately die. There are other insects whose period of existence is more prolonged and upon whose vital energies there are extraordinary demands—as in the production of locomotion—and who retain the voracity which characterized them in their larva state, yet whose bodies increase but little if any and whose existence also terminates very soon after sexual congress or deposition of ova, but whose lives are invariably prolonged when the sexes are kept apart. These facts considered conjointly establish beyond a doubt, first, that the act of generation is consummated not alone at the expense of organic compounds ingested during the larva or imago state, but also at the expense of constructive force which must result from a retrograde metamorphosis of either a portion of their organic compounds or organized tissue, one of which changes must be assumed as occurring in order to account for the relation of cause and effect which obviously exists between the death of the insect and the act mentioned; and second, that organic materials ingested which do not appear as accumulated organic compounds nor as living organized tissue, must be expressed either in a transmutation to constructive force or in exhaled carbonic acid and water, which is always proportionate to the work performed. And that the motor energy exhibited is not at the expense of organized tissue but at that of the organic compounds ingested, is manifest from the fact that the amount of food demanded and its metamorphosis, as indicated by the exhalation of carbonic acid and water, bears a direct proportional relation to the amount of power produced, while the metamorphosis of organized living tissue, indicated by the excretion of urea, bears no such proportional relation.

There remains for our examination another class of vital phenomena, namely, that which embraces those activities generated within nerve centres.

That the particular mode of force out of which these activities arise is motion, is obvious from the fact of its being propagated along the nerve substance from extremity to extremity, and as has been demonstrated by Bence Jones, and Emile Du Boise Reymond, at the rate of about nine-seven feet per second.

That nerve-force is as analagous to electricity as that force is to its correlate magnetism, is shown by the following facts, namely; that the transmission of a current of electricity along a nerve will cause contraction of the muscular fibers to which it leads; and that muscular contraction is excited also by diminishing the normal electrical current. For since contraction is the only possible mode by which muscular fibers can manifest their reception of stimuli to which they may be subjected, and since contraction does not occur from other causes than those mentioned, it follows, that nerve-force can be recognized only as a correlation of that form of physical force known as electricity. But electricity is a correlation of heat, and, as we have before shown, heat is a correlation of constructive force. What, then, is nerve-force but transmuted constructive force, released in those retrograde changes resulting from the functional activity of nerves and nerve centres.

But even the highest and most obscure manifestations of animal life have not escaped the revelations of modern science, and results of great value have been obtained which will not only help us in the solution of our present problem but through which, as I believe, we will yet be enabled to measure that most subtile and elusive of vital products, namely, thought. Every external manifestation of thought-force is a muscular one, as a word spoken or written, an expression of the face or a gesture. Hence this force must be intimately correlated to muscular motion.

It is after and only after the brain cell has been educated that thought-force, as expressed in muscular action, conveys meaning or excites concordant thoughts. Has it no other mode of expression? Can thought be manifested inwardly, without transformation into the actual energy of motion?

Experiments ingenious and reliable have answered these questions. Inasmuch as the methods for measuring electricity are very exact and the means for converting heat into electricity equally efficient, we may by the aid of a simple apparatus ascertain the measure of heat within the 0.004 of a degree. The apparatus consists of two small metallic bars for the conversion of heat and a delicate galvanometer for the measuring of the electrical product of the conversion.

Any change of temperature produced within the skull is soonest manifested externally in the depression located just above the occipital protuberance. If a pair of the bars just mentioned be fastened to the head at this point, and another pair, reversed in direction, to the arm or leg no deflection of the galvanometer will be produced by the electricity developed by a general rise of temperature since their equal and opposing currents will neutralize each other. If, however, from an increase of heat in that part to which one of these pairs of bars is attached, it be subjected to a higher temperature than its fellow, its increased electrical product will be immediately shown by the deflected needle.

By long practice a state of mental torpor can be induced lasting for hours, during which state no relative change of temperature can be observed in the parts of the body thus brought into comparison. But let a person knock at the door, or a single word be spoken, and though the experimenter remains otherwise absolutely passive, his reception of the intelligence causes the needle to swing through 20 degrees.

The analogue of this experiment is found in Heidenhain's Essay on Muscular Labor, in which the author established by repeated experiments, that when muscular contraction does not result in motion—as when one tries to raise a weight too heavy for him—the energy that would otherwise have appeared as motion takes the form of heat; and also in the fact, discovered by Emil Du Boise Reymond, that those molecular changes in the nerves which, if permitted to reach the muscular fibers, cause them to contract, if arrested in their progress may be transmitted into a deflection of the galvanometer—results which the law of correlation requires.

These experiments have proved also, that ideas which for a few moments affect the emotions only, produce more heat than hours of deep thought, from which fact it is evident that thoughts originated are such a portion of vital energy as escapes expression as heat.

Dr. Lombards's experiments show that the amount of heat developed by the recitation of emotional poetry aloud is in every case less than when the same compositions are recited to one's self; which fact is in accordance with the well known truth that emotion finds relief in physical demonstration, and also with the law of correlation. Nor does the doctrine of the correlation of the vital and physical forces rest on this class of evidence alone. The chemical products of mental activity appear to indicate that thought-force is evolved out of the organic compounds within our bodies and not at the expense of our organized tissues.

Dr. H. L. Wood in a valuable paper on the Influence of Mental Activity upon the Excretion of Phosphoric Acid by the Kidneys, published among the Proceedings of the Connecticut State Medical Society, has fully demonstrated that brain production, like that of the muscles, comes from the food and not from the disintegration of its own tissue. And how could this be true if thought-force was other than a transmutation of the energy which raised binary inorganic compounds to the higher chemical platform, represented in the daily food of man?

We observe then in conclusion, that a careful survey of the whole phenomena of life as exhibited in the two great natural kingdoms to which it belongs, reveals to us no instance or degree of vital action which is not the expression of some mode of that indefinable something to which we habitually refer all change, and which we agree in denominating—force.

The doctrine of the correlation of all terrestrial forces finds strong support in the unity of the source from which they alike arise. It is to the sun, as to the physical vicegerent of Deity, we must look for light and heat and all those subtler influences whose nature and operation are as yet but darkly understood. The winds, the rivers, the waves of the ocean and its profoundest currents are driven and maintained by the sun. All the power of steam is his. By the obscure processes of vegetable and animal vitality his power is stored away in various organic compounds, by the consumption of which alone life and activity are maintained; so that on close analysis we recognize the transformation of solar force, not only in our mechanical actions, the motions of our limbs and the sound of our voices, but even in the higher functions of thought and passion. As long as our functions are performed only by means of brain, nerve and muscle, so long it will be true that for all energy of thought, all intensity of feeling, all vigor of action of whatever kind we are ultimately dependent upon the sun.

The term for which I was chosen your President is about to expire. I thank you for your kindness both in choosing me as your presiding officer, and for your support while I have discharged his duties. With heartfelt wishes that your Society in future may be prosperous, and that you as individuals may be the recipients of those blessings which crown the good physician I conclude my duties.

